# Using ActionADE to create information continuity to reduce re-exposures to harmful medications: study protocol for a randomized controlled trial

**DOI:** 10.1186/s13063-021-05061-7

**Published:** 2021-02-05

**Authors:** Jeffrey P. Hau, Penelope M. A. Brasher, Amber Cragg, Serena Small, Maeve Wickham, Corinne M. Hohl

**Affiliations:** 1 Department of Emergency Medicine, University of British Columbia, 855 West 12th Avenue, Vancouver, British Columbia V5Z 1M9 Canada; 2 Centre for Clinical Epidemiology & Evaluation, Vancouver Coastal Health Research Institute, 828 West 10th Avenue, Vancouver, British Columbia V5Z 1M9 Canada; 3 School of Population and Public Health, University of British Columbia, 2206 E Mall, Vancouver, British Columbia V6T 1Z9 Canada; 4 Vancouver General Hospital, 855 West 12thAvenue, Vancouver, British Columbia V5Z 1M9 Canada

**Keywords:** Adverse drug events, Medication safety, Randomized controlled trial, Health information technology

## Abstract

**Background:**

Repeat exposures to culprit medications are a common cause of preventable adverse drug events. Health information technologies have the potential to reduce repeat adverse drug events by improving information continuity. However, they rarely interoperate to ensure providers can view adverse drug events documented in other systems. We designed ActionADE to enable rapid documentation of adverse drug events and communication of standardized information across health sectors by integrating with legacy systems.

We will leverage ActionADE’s implementation to conduct two parallel, randomized trials: patients with adverse drug reactions in the main trial and those diagnosed with non-adherence in a secondary trial. Primary objective of the main trial is to evaluate the effects of providing information continuity about adverse drug reactions on culprit medication re-dispensations over 12 months. Primary objective of the secondary trial is to evaluate the effect of providing information continuity on adherence over 12 months.

**Methods:**

We will conduct two parallel group, triple-blind randomized controlled trials in participating hospitals in British Columbia, Canada. We will enroll adults presenting to hospital with an adverse drug event to prescribed outpatient medication. Clinicians will document the adverse drug event in ActionADE. The software will use an algorithm to determine patient eligibility and allocate eligible patients to experimental or control. In the experimental arm, ActionADE will transmit information to PharmaNet, where adverse drug event information will be displayed in community pharmacies when re-dispensations are attempted. In the control arm, ActionADE will retain information in the local record. We will enroll 3600 adults with an adverse drug reaction into the main trial. The main trial’s primary outcome is re-dispensation of a culprit or same-class medication within 12 months; the secondary trial’s primary outcome will be adherence to culprit medication. Secondary outcomes include health services utilization and mortality.

**Discussion:**

These studies have the potential to guide policy decisions and investments needed to drive health information technology integrations to prevent repeat adverse drug events. We present an example of how a health information technology implementation can be leveraged to conduct pragmatic randomized controlled trials.

**Trial registration:**

ClinicalTrials.gov NCT04568668, NCT04574648. Registered on 1 October 2020.

## Introduction

### Background and rationale

Medication use is rising due to an aging population and expanding treatment indications for chronic diseases [1]. Simultaneously, adverse drug events—harmful and unintended events related to medication use or misuse—have increased [2–4]. In Canada, adverse events to outpatient medications cause over two million emergency department visits and 700,000 hospital admissions, costing over $1 billion in healthcare expenditures annually [4, 5]. Optimizing the benefits of medications while limiting their potential for harm is a public health priority across patient populations, health settings, and medical disciplines [6, 7].

Interventions have been implemented to reduce adverse drug events based on the premise that most result from errors [8]. This has led to a focus on reducing errors by ensuring the “five rights” of medication administration: giving the *right* drug to the *right* patient in the *right* dose by the *right* route at the *right* time. Many jurisdictions have implemented closed loop medication administration systems and drug-drug interaction software to ensure these *five rights*. While many medication safety technology evaluations have demonstrated reductions in errors that may have resulted in fewer potential adverse drug events (e.g., avoidance of co-prescription of two interacting medications), few have demonstrated reductions in *actual* adverse drug events (e.g., drug interaction resulting in patient harm) or benefits in patient-oriented outcomes [9, 10].

Robust prospective data help explain why: only 0.3% of clinically significant adverse drug events to outpatient medications were caused by errors in multi-center studies [3, 4, 11, 12]. In contrast, 33% of patients presenting to hospital with an adverse drug event due to outpatient medications were suffering an event caused by re-exposure to culprit medication—a medication that had previously caused harm [13]. In a Dutch study of seniors admitted to hospital because of an adverse drug reaction, a subset of adverse drug events defined as undesirable effects due to medication use within the therapeutic dosing range, 27% were restarted on the culprit drug within 180 days [14]. An Ontario study estimated that 54% of seniors admitted to hospital for hypoglycemia while on glyburide (contraindicated in seniors) or for a fall while on atypical neuroleptics or benzodiazepines were re-exposed within 180 days [15]. Thus, system-level interventions should focus on individuals who have experienced an adverse drug event and are at risk of unintentional re-exposure to that medication or medication class.

The second most common subset of adverse drug events to outpatient medications was caused by patient non-adherence [3, 4, 11, 12]. Non-adherence has been associated with increased downstream health services utilization and costs of care, likely reflective of worse patient outcomes [16]. It is unknown to what extent communication about non-adherence to outpatient medications can assist the care team in reinforcing patient adherence.

Patients with adverse drug events—whether they are adverse drug reactions or due to non-adherence—often seek care in hospitals due to the unexpected and serious nature of these events. After assessment and treatment, patients are discharged back into the care of a community-based provider who often cannot access the hospital’s medical record, may not receive a legible or detailed discharge summary and is at risk of either re-starting the culprit medication for chronic disease management in the case of an adverse drug reaction [4, 17–19]. If the adverse event was due to non-adherence, community-based care providers risk managing the patient without knowing about the patient’s non-adherence, and inappropriately up-titrating medication doses, or simply missing an opportunity to emphasize the importance of adherence. We developed ActionADE to address this type of information discontinuity.

### ActionADE

Prior to its design, we completed a systematic review of existing adverse drug event reporting systems and used ethnographic workplace observations and participatory methods to understand clinician workflow, barriers to adverse drug event documentation, and map the information systems with which ActionADE would need to be integrated with [20–22]. We then designed a stand-alone web-based user interface using agile design methods allowing users to document adverse drug events [20, 23]. After piloting and refining, we integrated ActionADE with the PharmaNet database. PharmaNet is a secure province-wide network that links all pharmacies in British Columbia to a central data system. In 2019, we integrated ActionADE uni-directionally with PharmaNet, allowing users to pull in demographic information and visualize their patient’s medication dispensing history [24, 25]. Since then, hospital pharmacists are using information recorded in ActionADE to report adverse drug reactions to Health Canada to meet a new federal adverse drug reaction reporting requirement (Bill C-17) [26–28]. In 2020, we enabled bi-directional integration with PharmaNet allowing clinicians to transmit standardized adverse drug event information back to the PharmaNet database. The three dominant community pharmacy systems in the geographic area of the trial will display the adverse drug event information in their systems and generate patient-specific medication-level alerts when pharmacists attempt to re-dispense a culprit medication. We will leverage ActionADE’s implementation to conduct two randomized controlled trials.

## Objectives

The primary objective of our main trial is to evaluate the effect of providing information continuity about adverse drug reactions using ActionADE on culprit drug re-dispensations over 12 months compared to standard care. The primary objective of the secondary trial will be to collect preliminary information about the provision of non-adherence information via ActionADE on subsequent adherence to the same medication over 12 months compared to standard care. Secondary objectives for both trials are to evaluate the effect on outpatient and emergency department visits, admissions, hospital days, and mortality.

## Trial design

This protocol describes two parallel-group, triple-blind randomized controlled trials among adults presenting to participating hospitals with an adverse drug event to a prescribed outpatient medication. Recruitment will occur over 18 months with a target sample size of 3600 for our main trial.

## Methods

### Study setting

The trials will take place in two urban tertiary care (Vancouver General and Saint Paul’s Hospitals) and one urban community hospital (Lions Gate Hospital) within the Greater Vancouver area, in British Columbia, Canada. Other hospitals may be added to accelerate recruitment into the trial if approved by the BC Ministry of Health.

### Eligibility criteria

The target population is adult (> 18 years) patients presenting to the Emergency Department with an adverse drug event to a prescribed outpatient medication. The sample population is patients with an adverse drug event that was reported in ActionADE. We will enroll patients diagnosed with adverse drug reactions in the main trial, and those diagnosed with non-adherence in a secondary trial. We will define adherence as individuals who meet the proportion of days covered threshold of 80% or greater (> 80% of prescribed medication doses within 12 months, based on the recorded medication dose and frequency, and the volume and dates of re-dispensations) [29].

Patients whose adverse drug event is categorized as life threatening will be excluded. We will exclude patients without a Provincial Health Number as this will prevent linkage with PharmaNet and other administrative data for outcomes ascertainment. We will also exclude patients diagnosed with adverse drug events to culprit medications not on the provincial formulary, as we will not be able to ascertain re-dispensations outcomes for these medications using PharmaNet data.

**Fig. 1:**
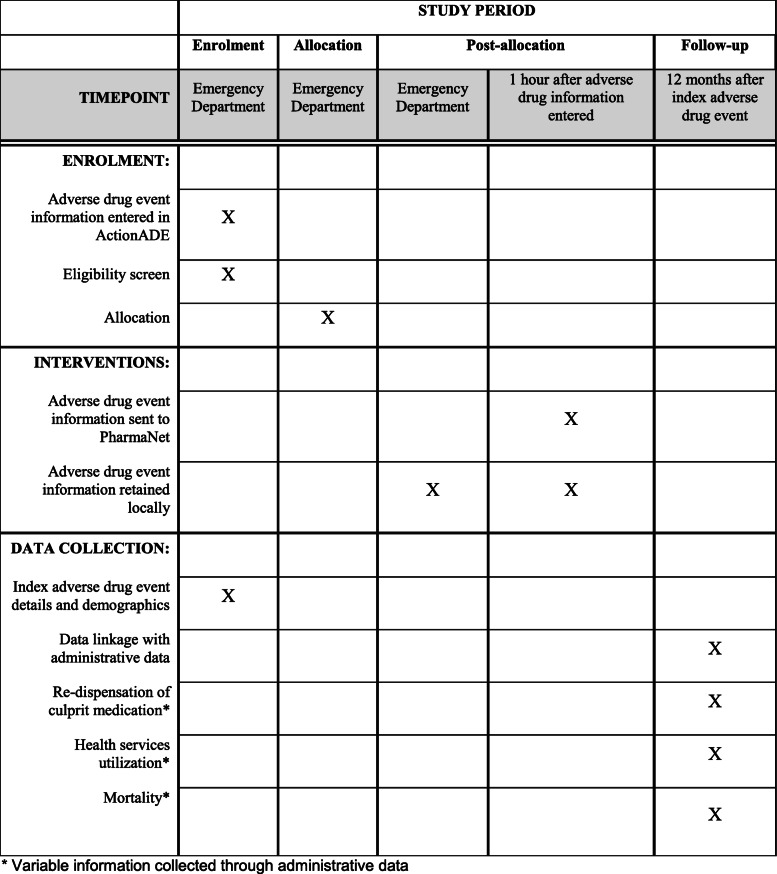
Standard Protocol Items: Recommendations for Interventional Trials diagram. Asterisk indicates variable information collected through administrative data

Figure 1 shows the SPIRIT (Standard Protocol Items: Recommendations for Interventional Trials) diagram for the trail procedures. Clinical pharmacists and physicians will document adverse drug events into ActionADE as part of routine clinical care. ActionADE will use an automated algorithm to determine each patient’s eligibility criteria for participating in the main and secondary trials.

### Recruitment and randomization

We will enroll consecutive eligible patients. Randomization is implemented in the ActionADE application. Randomization will be equal (1:1) and stratified by site and age (< 80, ≥ 80). A statistician otherwise uninvolved in the study will generate a list of treatment assignments using permuted blocks of varying sizes for each stratum. ActionADE will store the randomization list for each stratum. Once eligibility has been determined, the application will allocate the patient to the next available assignment within the stratum. For patients randomized to the experimental arm, the information will be pushed to PharmaNet to enable electronic information continuity. For patients randomized to control, the adverse drug events will be stored on a local server.

### Blinding

Treatment allocation is concealed through use of the application. Only ActionADE’s developer will have access to the randomization sequence. Care providers, study participants, outcomes assessors, and the analyst/statistician will be blinded to treatment arm. Community pharmacists cannot be blinded to the intervention, as we are studying the change in pharmacists’ dispensing behavior due to the provision of new adverse drug event information. The information will only appear for patients in the experimental arm.

Potential for unblinding and mitigation: If clinicians in hospital were to access a patient’s PharmaNet record after an adverse drug event was recorded, hospital providers could become unblinded as they will see whether or not the patient’s adverse drug event information appears in PharmaNet. For patients randomized to the control arm, the clinician could be more diligent with regard to the provision of information to the patient or the family physician diluting the treatment effect. We will mitigate the occurrence of unblinding in two ways: currently, the patient’s PharmaNet profile is downloaded and printed by clerks when the patient arrives in the Emergency Department, such that care providers already have access to the medication dispense history, making providers less likely to access PharmaNet for the dispense history. Second, we will build a 1-h delay from adverse drug event reporting to transmission of the information to PharmaNet in the experimental arm. We will review the time log within ActionADE to track the number of times and users who attempt to re-access PharmaNet using ActionADE after an adverse drug event report was created. If we see this behavior occurring using ActionADE, we will ask those users to avoid re-accessing PharmaNet. Finally, it is possible that pharmacists will change their behavior in all patients, because they may become more sensitized to the issue of adverse drug events. We believe this risk is small, because pharmacists are already used to collating adverse drug event information from their patient history and their own pharmacy records. If this occurs, this would dilute the treatment effect.

### Experimental intervention: information continuity

Patients in the experimental arm will have standardized adverse drug event information documented in ActionADE transmitted to and stored in PharmaNet. The adverse drug event information will become visible to any subsequent healthcare provider who accesses the patient’s PharmaNet profile. Community pharmacy software will import the adverse drug event information such that community pharmacists can view the adverse drug event information prior to dispensing medications.

Three community pharmacy software systems (Shoppers Drug Mart, ARI and Telus-Health/Kroll) dominate the community pharmacy market in the Greater Vancouver area and will cover > 90% of dispensations based on the BC Ministry of Health’s estimates (unpublished data). In each of those systems, the adverse drug event data will be displayed differently. In all systems, the data will generate pop-up alerts when community pharmacists attempt to re-dispense a culprit or same-class medication. However, only Telus-Health/Kroll will use forcing functions requiring community pharmacists to manually override alerts (while documenting the reason for the override) if they wish to re-dispense a culprit, or a same-class medication. Adherence information will be visible in all three systems, but will not generate pop-up alerts.

### Control group: standard care

Patients in the control group will have their adverse drug event information recorded in ActionADE, and their information will be retained locally, as is the current standard of care. This means that their adverse drug event information will not be visible to other providers via PharmaNet.

### Strategies to improve adherence to interventions

As patient enrolment and delivery of the intervention will be fully automated once an adverse drug event is docuemented in ActionADE, we do not anticipate any problems related to adherence with the allocated intervention or modification of the intervention. We have been piloting ActionADE in a participating emergency department for over 12 months with documentation of an average of 29.2 adverse drug events every month, with increasing use since December 2019 due to the newly passed Vanessa’s Law which mandates adverse drug reaction reporting in Canada [27, 28]. We will expand ActionADE’s use to additional sites according to the recruitment rate that we will monitor on a monthly basis. We will also promote its ongoing use through extensive end-user engagement in one-on-one onboarding and follow-up sessions, Rounds presentations and presentations at morbidity and mortality rounds, as well as incentive draws. Even though we believe the risk of non-adherence with adverse drug event reporting is small, we will track reporting in a planned mixed-methods implementation process evaluation which will include tracking recruitment of new users and sustained users on a monthly basis. Our planned process evaluation will capture barriers and contextual factors in ActionADE’s implementation should we need to troubleshoot.

### Relevant concomitant care permitted during the trials

All patients, irrespective of treatment arm allocation, will continue to have information about adverse drug events and medication contraindications incorporated into written emergency department, consultations, and medical progress notes, as well as into discharge summaries as is the current standard. Care providers can print out reports they generate in ActionADE or copy any documentation from the chart that they would normally provide to their patients as per their current practice. All patients will be able to request copies of their information at any time. All participants will receive standard medical care during the index emergency department visit and hospitalization.

### Outcomes

The primary outcome for our main trial will be culprit or same-class medication re-dispensing during follow-up. In our secondary trial, the primary outcome will be adherence to culprit medications over 12 months. Secondary outcomes for both trials will be the number of outpatient and emergency visits, hospital admissions, hospital bed days, and mortality during the follow-up period.

### Outcome ascertainment

Community pharmacists must enter all outpatient dispensed medications into PharmaNet for billing purposes. Therefore, outpatient medication dispensing data are considered accurate and complete in British Columbia with exceptions of samples provided by physicians and medications excluded from the provincial formulary (which are excluded from the trial). We will capture the generic names of patients’ dispensed medications, prescribed doses, routes and frequencies, and the dates and volumes of dispensations through patient-level linkage of study data with PharmaNet data using Personal Health Numbers, a unique lifetime identifier for health care in British Columbia. This will allow us to ascertain both re-dispensation outcomes for our main trial and adherence for our secondary trial.

We will capture inpatient and outpatient health services utilization and mortality by linking the study data with the Discharge Abstract Database, Medical Service Plan Client Registry, and Vital Statistics after the last recruit completes their 12 month follow-up period. All required data are available through Population Data BC and provide a uniform source of events that are reliably captured in British Columbia.

### Sample size

Based on our pilot work and the emergency department volumes of participating sites, we anticipate recruiting at least 200 patients per month, yielding a minimum of 3600 patients over an 18 month recruitment period. A previous study found that 26.6% (95% CI 17.3% to 38.5%) of seniors were re-prescribed a culprit medication within 6 months after an adverse drug reaction-related hospitalization [30]. An Ontario database study estimated that 54% of elderly patients were re-exposed to a high-risk medication within 6 months of a presentation related to an adverse drug reaction [31]. From our prior work, we anticipate that over one third of our patients will be seniors ≥ 80 years of age [13]. Assuming a re-exposure proportion of 30% in patients ≥ 80, and 15% in younger patients, we anticipate 20% of patients being re-exposed to culprit medications over a 12-month period in the control arm. With 3600 patients, we will have > 95% power to detect a 5% absolute reduction (from 20 to 15%) in culprit medication re-dispensing assuming *α* = 0.05 (2-sided) and approximating the analysis with a test of two proportions (Z-test) with no continuity correction. Through discussion with other clinicians, we believe that an absolute difference of 5% would be the minimal clinically important difference to change practice. Based on a prior study, we anticipate less than 1% loss to follow-up within 12 months due to unresolved linkages between Medical Service Plan and PharmaNet data. Although a total sample size of ~ 2000 would be adequate (> 80% power) for the primary analysis, we chose to recruit additional patients to allow for exploration of potential treatment effect modification due to the absence of alert overrides in one of the three community pharmacy systems.

Based on prior estimates of the proportion patients with adverse drug events who are categorized as being due to non-adherence, we anticipate approximately 2000 patients with an index event of non-adherence to accumulate over that time for our secondary trial [4, 32–34]. We will have at least 80% power to detect risk ratios of 0.70 to 0.85 over a range of non-adherence proportions (0.2 to 0.5).

### Data collection

All data required for the trial will be collected using ActionADE and through linkage to administrative data. After a provider logs on and enters a patient’s Provincial Health Number, ActionADE will pre-populate with demographic and medication information from PharmaNet. The clinician will add adverse drug event details using standardized and piloted drop-down menus.

### Ethical considerations and informed consent

The University of British Columbia Reserach Ethics Board has approved this protocol (H018-1332) and provided a waiver for obtaining informed consent as this trial meets the Tri-Council Policy Statement (TCPS) minimal risk criteria [35].

The rationale for requesting a waiver for informed consent is that we seek to evaluate the effectiveness of a potentially sustainable public health intervention that is being introduced by the BC Ministry of Health as a new part of routine care. The process of gaining informed consent would likely influence patient and provider behavior, which could negatively impact the trials’ internal validity. Patients would likely be hypersensitized to the issue of adverse drug events, which could lead to increased reporting of this information to subsequent care providers. This could lead to a dilution of the intervention effect, while not reflecting patients’ usual behaviors in the main trial. On the other hand, patients diagnosed with non-adherence could overreport their adherence behavior due to the stigma associated with non-adherence. This would put them at greater risk of overly aggressive medical management (e.g., inapprorpiate uptitration of medications) based on false reports of adherence putting them at greater risk for adverse drug reactions. For these reasons, we felt it was imperative that when evaluating ActionADE, participant behaviors mirror their behaviors in real-life [36].

### Data management and security

ActionADE stores data on a secure server administered by the provincial health authority. Data storage on the health authority’s server meets all the security and privacy regulations required in British Columbia. The local health authority and the BC Ministry of Health strictly regulate access to the ActionADE server. All patient identifiable fields in the system are encrypted with a master encryption key. This master cipher key itself is encrypted within a key vault, and there is no known practical computational method to decipher the patient identifiable data. ActionADE’s developer also has access to the ActionADE software for its refinement and technical support. The research team may need to access patient-level information as part of the metrics reporting and process evaluation. ActionADE’s developer and the study principal investigators have, and will maintain, a list of the employees of the developer who may have to access these data, all of which have all signed confidentiality agreements. The Vancouver Coastal Health Data Release Management Office has completed a Privacy Impact Assessment, and the health authority Data Stewardship Committee has approved ActionADE’s implementation.

At the beginning and end of the trial, we will transmit Provincial Health Numbers and demographic data for all enrolled patients from ActionADE to Population Data BC for linkage with administrative data. Population Data BC will strip the study patient identifiers and Provincial Health Numbers from the linked data set and assign a unique de-identify these data, and return them with linked de-identified administrative data. The research team members will access these data through Population Data BC’s Secure Research Environment to monitor and evaluate the trials.

### Statistical analysis

#### Main trial

All analyses will be by intention-to-treat. The between-group difference in the proportion of patients with a re-dispensing of the culprit medication within 12 months will be estimated using competing risk regression. Death will be treated as a competing risk; the regression will be stratified by hospital site and age category. In a secondary analysis, we will assess the sensitivity of the treatment estimate to the inclusion of potential confounders to the model: sex, adverse drug event severity, degree of certainty of the adverse drug event (e.g., suspect versus certain), and place of residence.

#### Secondary trial

We will evaluate the primary outcome of the secondary trial, adherence to the culprit medication, on an intention-to-treat basis. Patient adherence will be calculated as the ratio of the number of days medications were supplied by the pharmacist, based on the dispense history in PharmaNet, over the number of days until the next prescription refill for a 12-month period before and after the index visit. Adherence will be analyzed using a generalized linear model with gamma distribution and log link. Adherence will also be reported as ≥ 80% versus < 80%.

#### Sub-group analyses

The assessment for treatment effect modification due to the absence of alert overrides will be explored by including an alert type-by-treatment term in the primary model. Similarly, to explore the possibility of effect modification by age and sex, we will add age group-by-treatment and sex-by-treatment terms to the primary model. Prior to completion of recruitment a detail statistical analysis plan will be developed for secondary and exploratory analyses.

### Oversight and monitoring

The documentation, transfer of information, and care of individuals in the control group is unchanged from the current standardized procedures. We believe patients in the experimental arm may be more likely to have their medications permanently withdrawn than patients in the control group. However, both removal and re-exposure to culprit medications can lead to adverse outcomes, both of which are unavoidable risks that occur in routine medical practice. While patients in the control group may be at higher risk of experiencing repeat exposures to culprit medications which may result in repeat adverse drug events, they may also be more likely to remain on first-line treatments and as a result experience better disease-related outcomes. There is clinical equipoise about which of these risks are greater to participants. We anticipate no negative impact on the physical, mental, or spiritual health of participants, nor on their physical, economic, or social circumstances by participating in the trial.

A data safety monitoring board (DSMB) will monitor the study for mortality or any other poor health outcomes attributed to adverse drug events that are brought to the attention of the research team. The DSMB is an independent committee that will comprise a senior practicing emergency physician, a health services researcher, a statistician, and also a patient partner who are otherwise uninvolved in the study. The responsibilities of the DSMB are as follows: assessing any on-going reporting instances of adverse events which are attributable to the on-going trial and reported to the research team and monitoring whether recruitment and delivery of the intervention is progressing as expected to ensure adequate enrolment of patients. The time required for the linkage with administrative data precludes an interim analysis for efficacy. We will use the intervention codes “AI” (=adverse drug event Alert Inappropriate) and “AE” (=adverse drug event Data Entry Error) entered by community pharmacists in community pharmacy software as metrics to monitor patient safety, as those codes may indicate that pharmacists are not understanding or misinterpreting the adverse drug event information. The DSMB will monitor and discuss the use of AI and AE codes. If required, DSMB members will review the research and medical records of those patients in whom these codes were used to ensure the safety of the intervention.

### Dissemination plans

We will update the ClinicalTrials.gov database during, and after the completion of the study. We will also disseminate data and results of the trial through publications in peer-reviewed journals and conference presentations.

## Potential impact

Estimates suggest that repeat adverse drug events cause 65,358 Emergency Department visits and 24,117 hospitalizations in British Columbia annually [4, 16, 37]. ActionADE has the potential to improve healthcare quality by preventing unintentional re-exposures to harmful medications. This may reduce repeat adverse drug events, and adverse drug event-related outpatient and emergency department visits and hospital admissions, thereby enhancing health system sustainability. Modeling worst and best-case scenarios, the potential cost-avoidance of the intervention is substantial in British Columbia, due to the high incidence of repeat adverse drug events in our health system.

The two pragmatic trials we present will evaluate the impact of ActionADE on re-exposures to culprit medications for the two most common types of adverse drug events encountered in clinical practice, adverse drug reactions and events due to non-adherence [4, 32–34]. If successful, we will conduct an economic evaluation using the trial data. We believe this work has the potential to advance patient care, improve population health, and may reduce healthcare costs while providing an example of successful cross sector collaboration of a research team, health authority, and the BC Ministry of Health to enable innovation, implementation, and integration of new health information technology.

The ActionADE randomized trials will provide an example of evaluating a complex health information technology implementation and integration using a pragmatic randomized trial design. To date, few health information technologies (HIT) have been designed to communicate adverse drug event information across health sectors (e.g., to other systems or to community pharmacies) [22, 38], and most have used pre-post designs for evaluation. These cannot control for important confounders and co-interventions or capture unintended effects. A recent systematic review revealed 51 published randomized trials used to evaluate HITs, most open-label, and evaluating the use of mobile phones, text messaging, tele-monitoring, computer interfaces, and electronic alerts to improve care in specific domains of health [39]. None evaluated the effect of creating interoperability, the ability to exchange and make meaningful use of information, between computer systems or software on health outcomes. This benefit is often assumed, while unintended effects may not be captured. The trials we propose will integrate randomization into a software that enables interoperation between health information technologies, and uses administrative data to evaluate the impact of the intervention on outcomes, providing an example of how pragmatic randomized trials can be seamlessly integrated into a new health information technology being adopted into clinical practice [40].

## Trial status

Recruitment for this study will start in January 2021 and will be completed on June, 2022. The current protocol is version 1.0 dated August 20, 2020. We will communicate any significant protocol modifications to relevant parties (i.e., clinical research boards, trial registries, journals).

## Data Availability

This study will be analyzed using publically available administrative health data. The datasets are available through PopData BC (https://www.popdata.bc.ca/).
